# Adverse drug reactions among children with tuberculosis in China: a multicentre study, 2017–2022

**DOI:** 10.1080/07853890.2026.2645734

**Published:** 2026-03-25

**Authors:** Yiqing Zhou, Yu Kan, Ping Liu, Hongmei Xu, Juan Ma, Haiyan Li, Xin Yu, Qingshan Cai, Man Tian, Qing Fang, Xianhui Zeng, Guangxu Yang, Tao Yang, Fei Wang, Bin Chen, Leonardo Martinez

**Affiliations:** a School of Public Health, Hangzhou Medical College, Hangzhou, China; b The Second Clinical Medical College, Zhejiang Chinese Medical University, Hangzhou, China; c Department of Tuberculosis, Shanghai Public Health Clinical Center, Fudan University, Shanghai, China; d Division of Infectious Diseases, Chongqing Medical University Affiliated Children’s Hospital, Chongqing, China; e Department of Infectious Diseases, Qinghai Province Women and Children’s Hospital, Xining, China; f Department of Pediatric Pulmonology, The Second Affiliated Hospital and Yuying Children’s Hospital of Wenzhou Medical University, Wenzhou, China; g Department of Tuberculosis, Suzhou No. 5 People’s Hospital, Suzhou, China; h Department of Tuberculosis, Hangzhou Red Cross Hospital, Hangzhou, China; i Department of Respiratory Medicine, Children’s Hospital of Nanjing Medical University, Nanjing, China; j Department of Pulmonary Medicine, The First Affiliated Hospital of Ningbo University, Ningbo, China; k Department of Infectious Diseases, Hainan Women and Children’s Medical Center, Haikou, China; l Tuberculosis Prevention Institute, Changchun Infectious Disease Hospital, Changchun, China; m Scientific Research Center, Zhejiang Sukean Pharmaceutical Co. Ltd., Hangzhou, China; n Department of Tuberculosis Control and Prevention, Zhejiang Provincial Center for Disease Control and Prevention, Hangzhou, China; o Infectious Disease Prevention and Control, Zhejiang Key Lab of Vaccine, Hangzhou, China; p Department of Epidemiology, Boston University School of Public Health, Boston, MA, USA

**Keywords:** Tuberculosis, children, adverse drug reactions, China, multicentre

## Abstract

**Objective:**

Although tuberculosis (TB) treatment, if adequately taken and adhered to, is highly effective. To investigate the occurrence, frequency and related factors of adverse drug reactions (ADRs) during anti-TB treatment in children in China.

**Methods:**

We conducted a multicentre study and collected data from 11 representative paediatric specialists and general hospitals offering anti-TB treatment. The outcome of interest was the occurrence of any ADRs. Multivariable logistic regression was performed to explore factors associated with ADRs and to identify groups at high-risk for ADRs.

**Results:**

In total, 482 patients were enrolled; of whom, 94 (19%) reported an ADR. The most common ADRs were blood system damage (24%) and gastrointestinal reactions (24%). Most ADRs occurred within the intensive treatment period and were of short duration. Children with severe TB or those treated with HRZ(E) + second-line drugs in the intensive phase were at higher odds of developing ADRs (OR = 2.44, 95% CI: 1.36–4.39, *p* = 0.003; OR = 3.70, 95% CI: 2.01–6.81, *p* < 0.001, respectively).

**Conclusions:**

ADRs are prevalent in Chinese paediatric TB patients, predominantly haematological or gastrointestinal, transient during the intensive phase. Severe disease and HRZ(E) + second-line regimens confer elevated risk, necessitating targeted surveillance and optimized paediatric therapies balancing efficacy and safety.

## Introduction

Paediatric tuberculosis (TB) is amongst the top 10 health-related causes of death globally [1]. Despite highly effective preventive treatments, the age-specific risk of TB after *Mycobacterium tuberculosis* infection is highest in young children (<5 years of age); mortality is common after developing TB in this age group [2–6]. In 2023, there were an estimated 1.3 million new cases in children aged 0–14 years [7]. Among the 30 countries with high TB burden, China ranks third in the estimated number of TB cases (∼740,000), of which children aged 0–14 account for approximately 5% of total cases [7].

For children diagnosed with TB, anti-TB treatment typically involves the use of isoniazid, rifampin, pyrazinamide and ethambutol during an intensive phase, followed by isoniazid and rifampin during a consolidation phase. Characteristics of anti-TB treatment include the use of a wide variety of drugs and long duration of medication; due to this, adverse drug reactions (ADRs) can occur. Common ADRs include TB-specific drug induced liver injury, renal function damage and gastrointestinal reactions, etc. These ADRs, at the slight level, bring about physical and psychological sufferings to patients. In serious circumstances, they may directly influence the patients’ compliance with and effectiveness of the treatment, resulting in treatment interruption. Furthermore, it is possible to increase the risks of recurrence and drug resistance, exerting a tremendous influence on the economic burden of the patients’ families [8–10]. For instance, gastrointestinal reactions can cause patients to experience nausea, vomiting, abdominal pain, bloating, loss of appetite and other conditions, which affect the patients’ nutritional intake and are not conducive to the recovery from the disease. Mild drug induced liver injury is manifested as a transient increase in transaminase levels, while severe cases can lead to liver failure and even threaten the patient’s life. Some patients are forced to discontinue anti-TB treatment as a result, which affects the overall treatment outcome [11]. In addition, because children are in a special stage of growth and development, their drug absorption, metabolism and elimination characteristics are different from adults, and ADR is also different from adults [12,13]. Therefore, more attention should be paid to the frequency and severity of ADR in children with TB, and timely intervention should be carried out for patients.

Although several studies have reported adverse drug events in adults treated with TB, few studies have thoroughly investigated adverse drug events in children on treatment. We aimed to explore the frequency and timing of these events in children diagnosed with TB in a multicentre study conducted throughout China.

## Methods

### Study design, setting and population

This was a multicentre study including paediatric TB cases from 11 representative paediatric TB hospitals throughout China (Northeast, East, South, Southwest and Northwest regions), including Shanghai, Jiangsu, Zhejiang, Hainan, Chongqing, Qinghai and Jilin. Children with TB (<15 years of age) treated at 11 hospitals between 2017 and 2022 and weighing up to 40 kg were included in the study. We additionally excluded neonates aged 1 month and under and weighing <4 kg at the time of admission. Children with TB were treated with at least three drugs, isoniazid, rifampin and pyrazinamide, at doses determined according to pre-treatment weight. This was followed by treatment with at least two drugs, H and R, during the continuation period.

### Data collection

We reviewed health records and collected data from several data systems including the Hospital Information System (HIS), the Laboratory Information System (LIS), Picture Archiving and Communication Systems (PACS), among others. Variables that were collected included sex, age, malnutrition, site, severity of TB, treatment dose (including those from isoniazid, rifampin and pyrazinamide), treatment therapy in the intensive phase, and combination medication. Data on ADRs including the type of ADR, occurrence time, duration and severity. ADRs were retrospectively collected from the hospital management system (HIS, LIS) across participating centres, with systematic extraction of data tailored to different ADR types: (1) clinical symptoms (e.g. nausea, vomiting, gastrointestinal discomfort, skin allergies, arthralgia and numbness) were extracted from physicians’ progress notes and medical records; (2) laboratory-confirmed ADRs (e.g. liver/renal function impairment, haematological abnormalities) were documented via review of full-course laboratory test results (e.g. liver enzymes, renal function panels and blood counts); and (3) specialized ADRs were verified through relevant diagnostic records (e.g. hearing tests, psychiatric assessment scales).

### Definition

We divided drugs into high-dose and non-high-dose groups according to daily dose per kilogram and daily maximum dose. The high-dose group was defined as at least one of the daily doses per kilogram (kg) or daily maximum dose exceeding the upper limit. The non-high-dose group was defined as daily dose per kg and daily maximum dose not exceeding the upper limit. In particular, the dosing details of H, R and Z were as follows: H had a daily dose of 10 (7–15) mg/kg, with a maximum daily dose of 300 mg for individuals weighing <50 kg and a conventional administration frequency of once daily; R was administered at 15 (10–20) mg/kg daily, with a maximum dose of 600 mg for those weighing <50 kg, with a once-daily regimen; Z had a daily dose of 35 (30–40) mg/kg, with no specified daily maximum dose for individuals weighing <50 kg, and a standard administration frequency of once daily. Severe TB was defined as TB except for the following: peripheral lymph node; intrathoracic lymph node TB, no airway obstruction; uncomplicated TB pleural effusion or oligotrophic nonvacua’s disease, confined to the lung lobes, without miliary changes [14]. And the age group classification in this study was also based on the WHO Consolidated Guidelines on Tuberculosis (Module 5: Management of Tuberculosis in Children and Adolescents, 2022): (1) young child: aged 0–4 years; (2) older child: aged 5–9 years; (3) young adolescent: aged 10–14 years [14].

The occurrence of ADRs of all anti-TB drugs in this study was recorded in accordance with the relevant contents of China’s Adverse Drug Reaction Report and Monitoring and Management Measures [15]. The types of ADRs observed included liver dysfunction, kidney dysfunction, blood system damage, ototoxicity, gastrointestinal reaction, visual impairment, arthralgia, hypothyroidism, psychiatric, gynecomastia, dermatological and peripheral neuropathy.

The criteria for judging the severity of ADRs during the application of anti-TB drugs were according to the Anti-tuberculosis Drug Adverse Reaction Treatment Manual, which was divided into mild, moderate and severe criteria according to the relevant criteria [16]. *Mild*: After the occurrence of ADRs, the symptoms were mild without serious damage to the body. The discomfort symptoms of the patients did not show any progress in the process of continuous medication, and there was no need to receive targeted treatment. *Moderate*: After the occurrence of ADRs, the patient had obvious damage to the body, and the important organs or systems of the patient were damaged by the drug, resulting in the change of the patient’s treatment plan; *Severe*: the patient had obvious body injury during the medication, and the patient’s life safety was threatened, resulting in the discontinuation of the patient’s treatment plan.

### Statistical analysis

Sociodemographic and clinical characteristics of the study population were summarized by frequency and proportion. The outcome of interest was the occurrence of any ADRs, while sex, age, malnutrition, site, severity of TB, dosage (including H, R and Z) and treatment therapy in the intensive phase, and combination medication were identified as potential risk factors. Odds ratios (ORs) were calculated to measure the association between risk factors and ADRs. Univariate and multivariate logistic regression were used for statistical analysis. All statistical tests were double-tailed and 95% confidence intervals were used to assess statistical significance. Statistical analysis was performed using R 4.4.1 statistical software (R Foundation for Statistical Computing, Vienna, Austria).

### Ethics approval and consent to participate

This study has been approved by the Ethics Committee of Zhejiang Provincial Center for Disease Control and Prevention (approval number: 2023-018-01). Since this study only collected medical record information and did not involve relevant personal information of patients, it will not cause risks or adverse effects on the rights and health of subjects, and an application for informed consent exemption has been submitted (Ethics Committee of Zhejiang Center for Disease Control and Prevention).

## Results

Of the 482 patients we included, 94 cases experienced ADR. The majority were male (*n* = 259, 54%) and the proportion of children aged 0–4 years old was 39% (*n* = 189). A small number of patients had extrapulmonary TB (*n* = 28, 6%). Sixty-four percent (*n* = 307) were severe. The duration of intensive treatment was 3.20 (IQR: 2.00–6.00) months and the duration of continuation treatment was 8.00 (IQR: 5.48, 10.50) months. A small number of patients were treated with high doses of isoniazid (5%, *n* = 23), rifampicin (2%, *n* = 10) and pyrazinamide (4%, *n* = 18). During intensive therapy, isoniazid exceeded the daily dose per kg or daily maximum dose in 23 cases, and the median dose was 16.67 (15.75–17.73) mg/kg in 16 cases exceeding 15 mg/kg. There were seven patients with a daily maximum dose of more than 300 mg, and the dosage was between 350 and 450 mg. Rifampicin exceeded the upper limit of daily dose per kg in 10 cases, and the median dose was 21.96 (21.43–27.06) mg/kg. Eighteen patients exceeded the upper limit of the daily dose per kg, and the median dose was 47.48 (42.86–50.00) mg/kg. Some (12%, *n* = 59) patients were treated with second-line drugs. The second-line drugs mainly used in the present study were linezolid and levofloxacin. A small number of children also received combination medication such as antibiotics, mainly including meropenem, azithromycin and cephalosporins (18%, *n* = 87). There were statistically significant differences between the two groups in the severity of TB, treatment options and combination medication (*χ*^2^ = 15.63, *p* < 0.001 for severity of TB; *χ*^2^ = 30.98, *p* < 0.001 for treatment options; *χ*^2^ = 8.01, *p* = 0.005 for combination medication) (Table 1). When stratified by age, children aged 0–4 and 5–9 years with severe TB had a significantly higher incidence of ADRs than those with non-severe TB (*p* = 0.002 and *p* = 0.019, respectively). Use of HRZ(E) + second-line drugs was also associated with a higher incidence of ADR in both age groups (both *p* < 0.001). Among 0–4-year-olds, high-dose H and use of combination medications were significantly associated with ADRs (*p* = 0.039 and *p* < 0.001, respectively; Table S1).

**Table 1.** Socio-demographic, clinical characteristics of the adverse drug reactions among paediatric tuberculosis patients in China, 2017–2022.

Variables	Total (*n* = 482)	No ADR (*n* = 390)	ADR (*n* = 92)	*p*
Sex				0.919
Male	259 (53.73)	210 (81.08)	49 (18.92)	
Female	223 (46.27)	180 (80.72)	43 (19.28)	
Age				0.941
0–4 years old	189 (39.21)	152 (80.42)	37 (19.58)	
5–9 years old	171 (35.48)	138 (80.70)	33 (19.30)	
10–14 years old	122 (25.31)	100 (81.97)	22 (18.03)	
Malnutrition				0.983
No	409 (84.85)	331 (80.93)	78 (19.07)	
Yes	73 (15.15)	59 (80.82)	14 (19.18)	
Site				0.865
PTB	454 (94.19)	367 (80.84)	87 (19.16)	
EPTB	28 (5.81)	23 (82.14)	5 (17.86)	
Disease severity				**<.001**
Non-severe	175 (36.31)	158 (90.29)	17 (9.71)	
Severe	307 (63.69)	232 (75.57)	75 (24.43)	
Isoniazid				0.251
Non-high dose	459 (95.23)	374 (81.48)	85 (18.52)	
High dose	23 (4.77)	16 (69.57)	7 (30.43)	
Rifampicin				0.631
Non-high dose	472 (97.93)	383 (81.14)	89 (18.86)	
High dose	10 (2.07)	7 (70.00)	3 (30.00)	
Pyrazinamide				1.000
Non-high dose	464 (96.27)	375 (80.82)	89 (19.18)	
High dose	18 (3.73)	15 (83.33)	3 (16.67)	
Treatment				**<.001**
HRZ (E)	423 (87.76)	358 (84.63)	65 (15.37)	
HRZ (E) + second-line drug (s)	59 (12.24)	32 (54.24)	27 (45.76)	
Combination medication				**0.005**
No	395 (81.95)	329 (83.29)	66 (16.71)	
Yes	87 (18.05)	61 (70.11)	26 (29.89)	

The bold values indicate that the differences are statistically significant.

Among children aged 0–4 years, those with severe TB were more likely to develop ADRs (OR = 4.07, 95% CI: 1.61–10.33, *p* = 0.003). High-dose isoniazid (OR = 6.02, 95% CI: 1.29–28.19, *p* = 0.023), high-dose rifampicin (OR = 6.62, 95% CI: 1.06–41.15, *p* = 0.043), combination medication (OR = 3.70, 95% CI: 1.74–7.87, *p* < 0.001) and the HRZ (E) + second-line drug(s) regimen (OR = 4.38, 95% CI: 1.84–10.47, *p* < 0.001) were all significantly associated with the risk of adverse reactions in this age group. In the 5–9 years old group, the ‘Severe’ severity of TB (OR = 2.86, 95% CI: 1.16–7.03, *p* = 0.022) was significantly associated with adverse reactions; and the HRZ (E) + second-line drug(s) regimen (OR = 7.07, 95% CI: 2.52–19.79, *p* < 0.001) was an important factor for the increased risk of adverse reactions in this group. In the 10–14-year-old group, after univariate logistic regression analysis of each variable, the *p* values corresponding to the crude ORs were all >0.05. Moreover, since the rifampicin dose was not high for all children aged 10–14, rifampicin was not included in the univariate analysis (Table 2).

**Table 2.** Univariable logistic analysis of the adverse drug reactions among paediatric tuberculosis patients by different age groups in China, 2017–2022.

Age groups	0–4 years old (*n* = 189)		5–9 years old (*n* = 171)		10–14 years old (*n* = 122)	
	Crude odds ratio	*p* Value	Crude odds ratio	*p* Value	Crude odds ratio	*p* Value
Sex						
Male	1.00 (reference)		1.00 (reference)		1.00 (reference)	
Female	0.99 (0.47–2.07)	0.971	1.00 (0.47–2.13)	0.995	1.18 (0.46–3.02)	0.727
Malnutrition						
No	1.00 (reference)		1.00 (reference)		1.00 (reference)	
Yes	1.98 (0.75–5.24)	0.167	0.21 (0.03–1.62)	0.134	1.13 (0.40–3.19)	0.825
Site						
PTB	1.00 (reference)		1.00 (reference)		1.00 (reference)	
EPTB	1.25 (0.33–4.80)	0.742	1.21 (0.24–6.10)	0.820	0.00 (0.00–Inf)	0.992
Disease severity						
Non-severe	1.00 (reference)		1.00 (reference)		1.00 (reference)	
Severe	4.07 (1.61–10.33)	**0.003**	2.86 (1.16–7.03)	**0.022**	2.02 (0.63–6.47)	0.236
Isoniazid						
Non-high dose	1.00 (reference)		1.00 (reference)		1.00 (reference)	
High dose	6.02 (1.29–28.19)	**0.023**	0.40 (0.05–3.24)	0.391	3.23 (0.51–20.62)	0.214
Rifampicin						
Non-high dose	1.00 (reference)		1.00 (reference)		–	
High dose	6.62 (1.06–41.15)	**0.043**	0.00 (0.00–Inf)	0.989	–	–
Pyrazinamide						
Non-high dose	1.00 (reference)		1.00 (reference)		1.00 (reference)	
High dose	0.82 (0.09–7.21)	0.855	1.21 (0.24–6.10)	0.820	0.00 (0.00–Inf)	0.991
Treatment						
HRZ (E)	1.00 (reference)		1.00 (reference)		1.00 (reference)	
HRZ (E) + second-line drugs	4.38 (1.84–10.47)	**<.001**	7.07 (2.52–19.79)	**<.001**	2.97 (0.89–9.97)	0.077
Combination medication						
No	1.00 (reference)		1.00 (reference)		1.00 (reference)	
Yes	3.70 (1.74–7.87)	**<.001**	0.98 (0.31–3.14)	0.975	1.49 (0.43–5.09)	0.527

The bold values indicate that the differences are statistically significant.

The most common ADRs were blood system damage and gastrointestinal reaction (Table 3), accounting for 24% of all ADRs, respectively, which could be observed in 6% of patients. Followed by anti-TB drug-induced liver injury (ATDILI) (4%), kidney dysfunction (3%) and dermatological (2%). ATDILI often appeared at 0.70 (IQR: 0.23–1.23) months of anti-TB treatment and lasted for 1.50 (IQR: 0.90–2.30) months; kidney dysfunction often appeared at 3.40 (IQR: 0.20–5.90) months and lasted for 3.10 (IQR: 1.20–8.20) months; blood system damage appeared at 2.95 (IQR: 1.00–5.20) months and lasted for 1.70 (IQR: 0.50–5.80) months; gastrointestinal reaction lasted for 1.00 (IQR: 0.58–2.75) months at 2.00 (IQR: 0.98–3.28) months; visual impact appeared at 1.00 (IQR: 1.00–4.00) months and lasted for 1.00 (IQR: 1.00–3.10) months; dermatological appeared at 1.00 (IQR: 0.10–1.00) months and lasted for 0.20 (IQR: 0.20–1.00).

**Table 3.** Spectrum, occurrence time and duration of adverse drug reactions among paediatric tuberculosis patients in China, 2017–2022.

Types of ADR	Number of ADR	% patients with ADR	% total ADR	Time of occurrence	Duration of adverse event
ATDILI	18	3.7	16.2	0.70 (0.23, 1.23)	1.50 (0.90, 2.30)
Kidney dysfunction	15	3.1	13.5	3.40 (0.20, 5.90)	3.10 (1.20, 8.20)
Blood system damage	27	5.6	24.3	2.95 (1.00, 5.20)	1.70 (0.50, 5.80)
Ototoxicity	2	0.4	1.8	–	–
Gastrointestinal reaction	27	5.6	24.3	2.00 (0.98, 3.28)	1.00 (0.58, 2.75)
Visual impairment	7	1.5	6.3	1.00 (1.00, 4.00)	1.00 (1.00, 3.10)
Arthralgia	1	0.2	0.9	–	–
Hypothyroidism	1	0.2	0.9	–	–
Psychiatric	2	0.4	1.8	–	–
Gynecomastia	0	0.0	0.0	–	–
Dermatological	9	1.9	8.1	1.00 (0.10, 1.00)	0.20 (0.20, 1.00)
Peripheral neuropathy	2	0.4	1.8	–	–
Total	111	23.0	100	1.30 (0.50, 4.68)	1.35 (0.60, 3.93)

Among patients included in the analysis, 79 (16%) and 10 (2%) participants experienced one or two ADRs, respectively. One patient (<1%) experienced three, four and five ADRs each, respectively.

**Figure 1.**
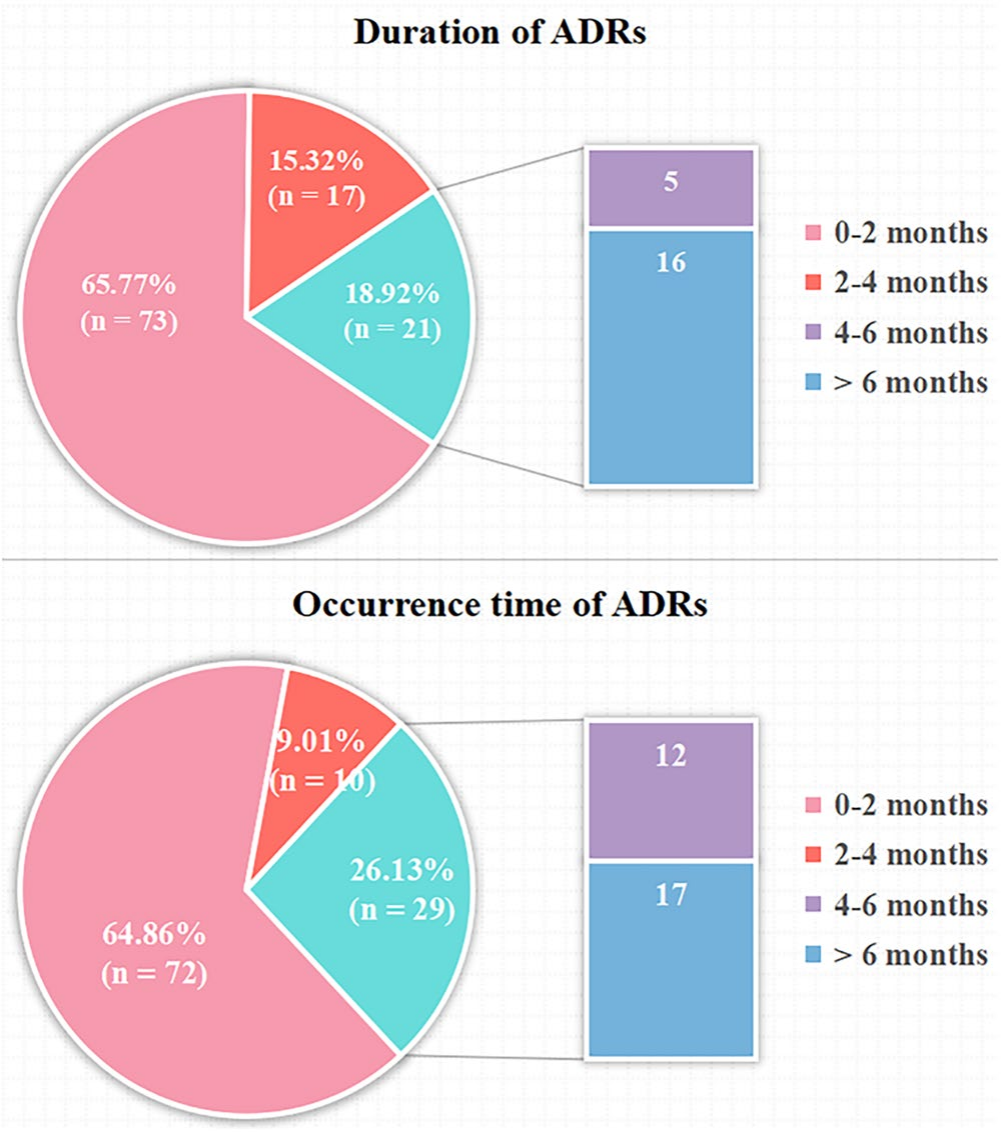
Occurrence time and duration of each ADR among paediatric tuberculosis patients in China, 2017–2022.

The median time and IQR of various ADRs were during the intensive period (Table 3, Figure 1); most (66%) of the ADRs were short-term (≤2 months) (Figure 1). Some (14%) of the ADRs were long-term (>6 months) and the details were as follows: there was one case of ATDILI (9 months), four cases of kidney dysfunction (8.2, 8.7, 11.1 and 12.3 months), five cases of blood system damage (11.0, 11.3, 12.3, 13.8 and 14.1 months), one case of gastrointestinal reaction (11.4 months), one case of visual impairment (9.1 months), one case of arthralgia (6.5 months), one case of hypothyroidism (13.0 months), one case of psychiatric adverse reaction (6.5 months) and one case of dermatological adverse reaction (10.0 months). The treatment plan and dosage in the continuous period may have little impact on the occurrence of ADRs, so only the impact of the treatment plan and dosage in the intensive period was analysed. Univariate logistic regression showed that disease severity, treatment and combination medication were the influencing factors of ADRs. After multivariable logistic regression, the results showed that the odds of ADRs in patients with severe TB were 2.39 times higher than those in non-severe patients (OR = 2.44, 95% CI: 1.36–4.39, *p* = 0.003); patients treated with HRZ(E) + second-line drug(s) in the intensive phase were at higher odds of developing ADRs compared to those treated with HRZ(E) alone (OR = 3.70, 95% CI: 2.01–6.81, *p* < 0.001) (Table 4).

**Table 4.** Factors associated with the occurrence of the adverse drug reactions among paediatric tuberculosis patients in China, 2017–2022.

Variables	Crude odds ratio	*p* Value	Adjusted odds ratio	*p* Value
Sex				
Male	1.00 (reference)		1.00 (reference)	
Female	1.02 (0.65–1.61)	0.919	1.05 (0.65–1.71)	0.838
Age				
0–4 years old	1.00 (reference)		1.00 (reference)	
5–9 years old	0.98 (0.58–1.66)	0.947	1.12 (0.64–1.97)	0.688
10–14 years old	0.90 (0.50–1.62)	0.735	0.97 (0.51–1.83)	0.917
Malnutrition				
No	1.00 (reference)		1.00 (reference)	
Yes	1.01 (0.53–1.90)	0.983	0.77 (0.38–1.54)	0.455
Site				
PTB	1.00 (reference)		1.00 (reference)	
EPTB	0.92 (0.34–2.48)	0.865	0.61 (0.21–1.84)	0.383
Disease severity				
Non-severe	1.00 (reference)		1.00 (reference)	
Severe	3.00 (1.71–5.28)	**<.001**	2.44 (1.36–4.39)	**0.003**
Isoniazid				
Non-high dose	1.00 (reference)		1.00 (reference)	
High dose	1.93 (0.77–4.82)	0.162	1.31 (0.45–3.88)	0.621
Rifampicin				
Non-high dose	1.00 (reference)		1.00 (reference)	
High dose	1.84 (0.47–7.27)	0.382	1.35 (0.27–6.83)	0.717
Pyrazinamide				
Non-high dose	1.00 (reference)		1.00 (reference)	
High dose	0.84 (0.24–2.97)	0.790	0.74 (0.19–2.90)	0.669
Treatment				
HRZ (E)	1.00 (reference)		1.00 (reference)	
HRZ (E) + second-line drug (s)	4.65 (2.61–8.27)	**<.001**	3.70 (2.01–6.81)	**<.001**
Combination medication				
No	1.00 (reference)		1.00 (reference)	
Yes	2.12 (1.25–3.61)	**0.005**	1.60 (0.89–2.89)	0.118

The bold values indicate that the differences are statistically significant.

**Figure 2.**
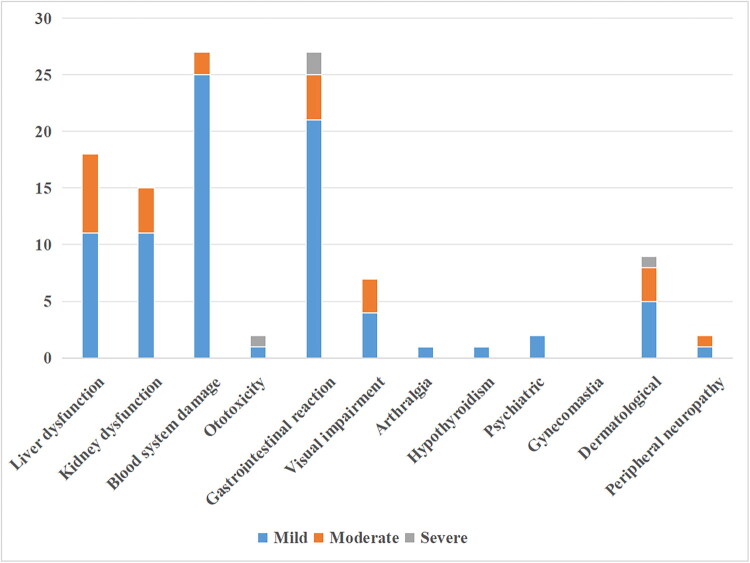
Severity assessment of each ADR among paediatric tuberculosis patients in China, 2017–2022.

Patients were more likely to have ATDILI and lead to protocol change than other ADRs (Figure 2), followed by kidney dysfunction, gastrointestinal reaction and visual impairment. Among the ADRs that led to treatment discontinuation, gastrointestinal reaction was the most common, followed by ototoxicity, and dermatological.

## Discussion

In a large hospital-based study of children with TB from 11 representative paediatric TB hospitals throughout China, the incidence of ADRs was common reaching almost one in five children. Although the incidence of ADRs in our study was lower than the reported range of ADRs among adults with TB [17–24], it is relatively similar to the incidence reported in some other paediatric TB studies [25–27]. In general, the incidence of ADR was high, and there were large differences between different studies, which might be related to a variety of factors. First of all, different studies had differences in criteria and features of included cases. Second, treatment regimens, types of monitoring, and patient management strategies varied. Since the TB diagnosis and treatment hospitals included in this study were basically provincial or municipal regional diagnosis and treatment centres, more difficult and critical TB cases would be treated than at grass-roots hospitals. In our study, the proportion of severe children with TB was higher, at 63.69%. This high proportion of severe cases may partially contribute to the relatively high incidence of ADRs observed in our study, given that severe cases often require more intensive or prolonged treatment regimens.

ADRs among paediatric TB patients during anti-TB treatment were predominantly haematological damage, gastrointestinal reactions, ATDILI and renal dysfunction. Specifically, ATDILI accounted for only 16% of all ADRs, which was significantly lower than the occurrence frequency reported in adult TB cases [28–30]. In terms of temporal distribution, 56% of paediatric liver injuries occurred within the first 2 weeks of treatment, earlier than the peak incidence at 1–2 months observed in adults [31]. Most ADRs in children with TB emerged during the intensive phase of treatment, consistent with findings in adult studies [29]. Regarding severity, only 17% of paediatric TB patients required drug discontinuation due to ADRs, primarily attributed to gastrointestinal reactions and ototoxicity. Notably, this rate remains substantially lower even when compared to the discontinuation rates observed in adults with multidrug-resistant TB – despite the fact that the latter population typically faces more complex treatment regimens and higher baseline risks of adverse reactions [32,33]. These findings suggested that children with TB had decent tolerance to anti-TB drugs. This provided a confident basis for clinicians to complete the treatment course through supportive therapies (such as hepatoprotective agents and antiemetics) rather than radical drug regimen changes in the presence of mild to moderate ADRs.

Gastrointestinal reactions caused by anti-TB drugs are relatively common in clinical practice, often occurring in the early stages of anti-TB treatment and manifesting as nausea, vomiting, chest burning sensation, etc. In this study, blood system damage accounted for about 1/4 of all ADRs. For ADRs in the blood system that occur during anti-TB treatment, the current recommendation is to take corresponding measures based on the severity of the damage. If symptoms are mild, anti-TB drugs can be continued while closely monitoring changes in blood counts. If the reaction is more severe, the drugs need to be discontinued until the blood routine test returns to normal, and then consideration can be given to restarting the drug treatment at a lower dose [34,35]. ATDILI accounted for about 1/6 of all recorded ADRs. Previous studies have shown that the occurrence of ATDILI is related to many factors, including host factors, drug factors and other factors [36–39]. However, the mechanism of ATDILI is still unclear, which is mainly related to abnormal drug metabolism, mitochondrial damage, immune function damage and genetic factors [40]. Therefore, in the process of anti-TB treatment, changes of liver biochemical indicators should be closely monitored. If the patient has ATDILI, timely intervention should be carried out to prevent serious progression and poor prognosis. Visual impairment is usually associated with ethambutol and linezolid, and the severity of the impairment is related to its dose and duration [41–43]. Relevant studies on TB in children have shown that the incidence of visual impairment related to ethambutol during anti-TB treatment was <1% [41,42]. Our results found that 1.5% of children had visual impairment. Clinically, ethambutol should be used with caution for infants and non-responders who cannot complain and cooperate with visual acuity examination.

Compared with adult TB, TB in children has atypical clinical manifestations, rapid disease progression and dissemination into severe TB [44,45]. Severe patients may have concurrent autoimmune diseases, and the weakened immune function leads to disorders in drug absorption and metabolism. These patients typically need a combination of multiple anti-TB drugs, or even higher doses or longer courses of treatment. This complex treatment regimen increases the risk of drug–drug interactions, thereby elevating the relative risk of ADRs. In addition, some severe patients may be complicated with autoimmune diseases, and the immune system function will decline, which will affect the metabolism and absorption of drugs and increase the risk of ADRs. Although HRZ is the recommended regimen for the intensive phase of TB in children, in clinical practice, doctors may adjust the medication regimen according to the actual situation of patients. For those patients with complex or severe conditions, second-line drugs may be used in combination at the beginning of treatment. Our results showed that these patients were more prone to ADR. This may be because some second-line drugs lack the recommended dose for children, which makes it difficult for clinicians to adjust the dose according to their weight [46,47]. Therefore, for children with TB, clinicians should be cautious in the use of first-line drugs combined with second-line drugs, and if used, the occurrence of ADR in children needs to be closely monitored and early intervention and treatment should be carried out.

Early and correct treatment of ADRs is the key to ensuring patient compliance and successful treatment. Therefore, medical personnel should pay attention to ADRs, explain the possible ADRs and their clinical manifestations to patients and their guardians in detail before anti-TB treatment, and guide patients to conduct self-monitoring to detect and deal with abnormalities in time. At the same time, it is necessary to strengthen the training of medical personnel on the types of ADRs and the risk factors of ADRs, so that they can master the principles and procedures of ADRs.

## Conclusions

ADRs are frequently observed during anti-TB treatment in children in China, with haematological system abnormalities and gastrointestinal disturbances being the most common manifestations. The majority of ADRs occur during the intensive phase of treatment and are transient in nature, although a subset may persist over a longer duration. Patients with severe forms of TB or those receiving intensified regimens combining HRZ(E) with second-line agents are at increased risk of developing ADRs. To improve treatment safety and patient adherence, it is recommended to implement targeted monitoring for ADRs – particularly among high-risk paediatric patients during the intensive treatment phase. Furthermore, there is an urgent need to develop optimized, child-appropriate therapeutic strategies that effectively minimize adverse events without compromising antimicrobial efficacy.

## Supplementary Material

Table_S1_Characteristics_of_the_adverse_drug_react clean.docx

## Data Availability

The data that support the findings of this study are available from the corresponding author [Fei Wang] upon reasonable request.

## References

[1] Dodd PJ, Yuen CM, Sismanidis C, et al. The global burden of tuberculosis mortality in children: a mathematical modelling study. Lancet Glob Health. 2017;5(9):e898–e906. doi:10.1016/S2214-109X(17)30289-9. 28807188 PMC5556253

[2] Pelzer PT, Stuck L, Martinez L, et al. Effectiveness of the primary Bacillus Calmette–Guérin vaccine against the risk of *Mycobacterium tuberculosis* infection and tuberculosis disease: a meta-analysis of individual participant data. Lancet Microbe. 2025;6(2):100961. doi:10.1016/j.lanmic.2024.100961. 39709975 PMC12778190

[3] Martinez L, Seddon JA, Horsburgh CR, et al. Effectiveness of preventive treatment among different age groups and *Mycobacterium tuberculosis* infection status: a systematic review and individual-participant data meta-analysis of contact tracing studies. Lancet Respir Med. 2024;12(8):633–641. doi:10.1016/S2213-2600(24)00083-3. 38734022 PMC12061052

[4] Du Preez K, Jenkins HE, Martinez L, et al. Global burden of tuberculous meningitis in children aged 0–14 years in 2019: a mathematical modelling study. Lancet Glob Health. 2025;13(1):e59–e68. doi:10.1016/S2214-109X(24)00383-8. 39706662 PMC11729397

[5] da Costa FBP, Nicol MP, Botha M, et al. *Mycobacterium tuberculosis* infection and tuberculosis disease in the first decade of life: a South African birth cohort study. Lancet Child Adolesc Health. 2024;8(12):891–899. doi:10.1016/S2352-4642(24)00256-6. 39515364 PMC11579303

[6] Martinez L, Le Roux DM, Barnett W, et al. Tuberculin skin test conversion and primary progressive tuberculosis disease in the first 5 years of life: a birth cohort study from Cape Town, South Africa. Lancet Child Adolesc Health. 2018;2(1):46–55. doi:10.1016/S2352-4642(17)30149-9. 29457055 PMC5810304

[7] WHO. Global tuberculosis report 2024. Geneva: World Health Organization; 2024. Available from: https://www.who.int/teams/global-tuberculosis-programme/tb-reports/global-tuberculosis-report-2024

[8] Koh Y, Yap CW, Li SC. A quantitative approach of using genetic algorithm in designing a probability scoring system of an adverse drug reaction assessment system. Int J Med Inform. 2008;77(6):421–430. doi:10.1016/j.ijmedinf.2007.08.010. 17921048

[9] Nagpal M, Devgun P, Kalra RK, et al. Study on the adverse effects affecting treatment outcome in smear positive tuberculosis patients under DOTS in Amritsar city. Int J Community Med Public Health. 2018;5(2):801. doi:10.18203/2394-6040.ijcmph20180272.

[10] Arbex MA, Varella M, Siqueira H, et al. Antituberculosis drugs: drug interactions, adverse effects, and use in special situations—part 1: first-line drugs. J Bras Pneumol. 2010;36(5):626–640. doi:10.1590/s1806-37132010000500016. 21085830

[11] Zhao Yanlin CM. Technical guidelines for tuberculosis prevention and control in China: technical guidelines for tuberculosis prevention and control in China in Chinese. 2021:05:388.

[12] Marais BJ, Gie RP, Schaaf HS, et al. The natural history of childhood intra-thoracic tuberculosis: a critical review of literature from the pre-chemotherapy era. Int J Tuberc Lung Dis. 2004;8(4):392–402. 15141729

[13] Shingadia D, Novelli V. Diagnosis and treatment of tuberculosis in children. Lancet Infect Dis. 2003;3(10):624–632. doi:10.1016/s1473-3099(03)00771-0. 14522261

[14] WHO. WHO consolidated guidelines on tuberculosis. Module 5: management of tuberculosis in children and adolescents. World Health Organization; 2022. 35404556

[15] Management measures for adverse drug reaction reporting and monitoring in Chinese. China Journal of Pharmaceutical Economics, 2011;2011(5):68–75.

[16] Donglou X, Yu M, Lizhen Z. Diagnosis and treatment manual for adverse reactions of anti-tuberculosis drugs in Chinese. People’s Medical Publishing House. 2009.

[17] Marra F, Marra CA, Bruchet N, et al. Adverse drug reactions associated with first-line anti-tuberculosis drug regimens. Int J Tuberc Lung Dis. 2007;11(8):868–875. 17705952

[18] Przybylski G, Dąbrowska A, Trzcińska H. Alcoholism and other socio-demographic risk factors for adverse TB-drug reactions and unsuccessful tuberculosis treatment – data from ten years’ observation at the Regional Centre of Pulmonology, Bydgoszcz, Poland. Med Sci Monit. 2014;20:444–453. doi:10.12659/MSM.890012. 24643127 PMC3965286

[19] Damasceno GS, Guaraldo L, Engstrom EM, et al. Adverse reactions to antituberculosis drugs in Manguinhos, Rio de Janeiro, Brazil. Clinics. 2013;68(3):329–337. doi:10.6061/clinics/2013(03)oa08. 23644852 PMC3611752

[20] Javadi MR, Shalviri G, Gholami K, et al. Adverse reactions of anti-tuberculosis drugs in hospitalized patients: incidence, severity and risk factors. Pharmacoepidemiol Drug Saf. 2007;16(10):1104–1110. doi:10.1002/pds.1468. 17823987

[21] Gholami K, Kamali E, Hajiabdolbaghi M, et al. Evaluation of anti-tuberculosis induced adverse reactions in hospitalized patients. Pharm Pract. 2006;4(3):134–138. PMC4156846 25214900

[22] Chhetri AK, Saha A, Verma SC, et al. Study of adverse drug reactions caused by first line anti-tubercular drugs used in directly observed treatment, short course (DOTS) therapy in Western Nepal, Pokhara. J Pak Med Assoc. 2008;58(10):531–536. 18998303

[23] Yagi M, Shindo Y, Mutoh Y, et al. Factors associated with adverse drug reactions or death in very elderly hospitalized patients with pulmonary tuberculosis. Sci Rep. 2023;13(1):6826. doi:10.1038/s41598-023-33967-6. 37100850 PMC10133295

[24] Maciel EL, Guidoni LM, Favero JL, et al. Adverse effects of the new tuberculosis treatment regimen recommended by the Brazilian Ministry of Health. J Bras Pneumol. 2010;36(2):232–238. 20485945 10.1590/s1806-37132010000200012

[25] Laghari M, Talpur BA, Syed Sulaiman SA, et al. Adverse drug reactions of anti-tuberculosis treatment among children with tuberculosis. Int J Mycobacteriol. 2020;9(3):281–288. doi:10.4103/ijmy.ijmy_75_20. 32862161

[26] Li Y, Zhu Y, Zhong Q, et al. Serious adverse reactions from anti-tuberculosis drugs among 599 children hospitalized for tuberculosis. Pediatr Infect Dis J. 2017;36(8):720–725. doi:10.1097/INF.0000000000001532. 28060046

[27] Abdusalomova M, Denisiuk O, Davtyan H, et al. Adverse drug reactions among children with tuberculosis in Tashkent, Uzbekistan, 2019. Int J Environ Res Public Health. 2021;18(14):7574. doi:10.3390/ijerph18147574. 34300026 PMC8308012

[28] Wang J. Analysis of adverse reactions to anti-tuberculosis drugs in multidrug-resistant pulmonary tuberculosis patients in this region from 2021 to 2023. Chin Health Care. 2025;43(4):109–111.

[29] Shangqing T, Liu Z, Song Z, et al. Analysis of epidemiological characteristics of adverse drug reactions caused by anti-tuberculosis drugs in a hospital. Anti-Infect Pharm. 2024;21(7):691–694.

[30] Shi C, Yang B, Yang J, et al. Evaluation of adverse reactions induced by anti-tuberculosis drugs among hospitalized patients in Wuhan, China: a retrospective study. Medicine. 2024;103(20):e38273. doi:10.1097/MD.0000000000038273. 38758847 PMC11098174

[31] Binbin C, Guangming X. Clinical features of drug-induced liver injury induced by anti-tuberculotic drugs. J Front Med. 2025;15(9):43–45.

[32] Haobin K, Shouyong T, Yuhong X, et al. Adverse drug reactions of chemotherapy and treatment in patients with multidrug resistant tuberculosis. J Tuberc Lung Health. 2015;4(4):219–222.

[33] Jian G, Peijun T, Jia L, et al. Analysis of occurrence and clinical characteristics of cycloserine-related adverse drug reactions in patients with multidrug-resistant tuberculosis. Anti-Infect Pharm. 2024;21(2):131–136.

[34] Nahid P, Dorman SE, Alipanah N, et al. Official American Thoracic Society/Centers for Disease Control and Prevention/Infectious Diseases Society of America Clinical Practice Guidelines: treatment of drug-susceptible tuberculosis. Clin Infect Dis. 2016;63(7):e147–e195. doi:10.1093/cid/ciw376. 27516382 PMC6590850

[35] Furin J, Seddon J, Perez-Velez C. Management of drug-resistant tuberculosis in children: a field guide. Boston (MA): Sentinel Project for Pediatric Drug-Resistant Tuberculosis; 2015.

[36] Sun Q, Zhang Q, Gu J, et al. Prevalence, risk factors, management, and treatment outcomes of first-line antituberculous drug-induced liver injury: a prospective cohort study. Pharmacoepidemiol Drug Saf. 2016;25(8):908–917. doi:10.1002/pds.3988. 26935778

[37] Lan Z, Ahmad N, Baghaei P, et al. Drug-associated adverse events in the treatment of multidrug-resistant tuberculosis: an individual patient data meta-analysis. Lancet Respir Med. 2020;8(4):383–394. doi:10.1016/S2213-2600(20)30047-3. 32192585 PMC7384398

[38] Zhang T, Ikejima T, Li L, et al. Impairment of mitochondrial biogenesis and dynamics involved in isoniazid-induced apoptosis of HepG2 cells was alleviated by p38 MAPK pathway. Front Pharmacol. 2017;8:753. doi:10.3389/fphar.2017.00753. 29123480 PMC5662931

[39] Sobhonslidsuk A, Poovorawan K, Soonthornworasiri N, et al. The incidence, presentation, outcomes, risk of mortality and economic data of drug-induced liver injury from a national database in Thailand: a population-base study. BMC Gastroenterol. 2016;16(1):135. doi:10.1186/s12876-016-0550-0. 27793116 PMC5084315

[40] Jaeschke H, Gores GJ, Cederbaum AI, et al. Mechanisms of hepatotoxicity. Toxicol Sci. 2002;65(2):166–176. doi:10.1093/toxsci/65.2.166. 11812920

[41] Donald PR, Maher D, Maritz JS, et al. Ethambutol dosage for the treatment of children: literature review and recommendations. Int J Tuberc Lung Dis. 2006;10(12):1318–1330. 17167947

[42] Thee S, Detjen A, Quarcoo D, et al. Ethambutol in paediatric tuberculosis: aspects of ethambutol serum concentration, efficacy and toxicity in children. Int J Tuberc Lung Dis. 2007;11(9):965–971. 17705973

[43] Frippiat F, Bergiers C, Michel C, et al. Severe bilateral optic neuritis associated with prolonged linezolid therapy. J Antimicrob Chemother. 2004;53(6):1114–1115. doi:10.1093/jac/dkh199. 15117929

[44] Bijker EM, Horn L, LaCourse S, et al. The inclusion of children and adolescents in tuberculosis diagnostic development and evaluation—a consensus statement. Lancet Infect Dis. 2024;24(11):e688–e695. doi:10.1016/S1473-3099(24)00339-6. 38971177

[45] Newton SM, Brent AJ, Anderson S, et al. Paediatric tuberculosis. Lancet Infect Dis. 2008;8(8):498–510. doi:10.1016/S1473-3099(08)70182-8. 18652996 PMC2804291

[46] Donald PR, Maritz JS, Diacon AH. The pharmacokinetics and pharmacodynamics of rifampicin in adults and children in relation to the dosage recommended for children. Tuberculosis. 2011;91(3):196–207. doi:10.1016/j.tube.2011.02.004. 21429802

[47] Chinese Society for Tuberculosis, Chinese Medical Association. Expert consensus on off-label use of antituberculosis drugs (2023 update). Chin J Tuberc Respir Dis. 2023;46(11):1085–1102. 10.3760/cma.j.cn112147-20230809-00062 37914420

