# Supplementary material for: Adverse drug reactions among children with tuberculosis in China: a multicentre study, 2017–2022
Source: Ann Med. 2026 Mar 25;58(1):2645734. doi: 10.1080/07853890.2026.2645734 (PMC13021018; doi:10.1080/07853890.2026.2645734)
Supplement: Table_S1_Characteristics_of_the_adverse_drug_react clean.docx [file IANN_A_2645734_SM9991.docx]

**Table S1 Characteristics of the adverse drug reactions among pediatric tuberculosis patients by different age groups in China, 2017-2022.**

| **Age groups** | **0-4 years old**  **(n = 189)** | | | **5-9 years old**  **(n = 171)** | | | **10-14 years old**  **(n = 122)** | | |
| --- | --- | --- | --- | --- | --- | --- | --- | --- | --- |
|  | **No ADR**  **(n = 152)** | **ADR**  **(n = 37)** | ***P*** | **No ADR**  **(n = 138)** | **ADR**  **(n = 33)** | ***P*** | **No ADR**  **(n = 100)** | **ADR**  **(n = 22)** | ***P*** |
| **Sex** |  |  | 0.971 |  |  | 0.995 |  |  | 0.727 |
| Male | 94 (80.34) | 23 (19.66) |  | 71 (80.68) | 17 (19.32) |  | 45 (83.33) | 9 (16.67) |  |
| Female | 58 (80.56) | 14 (19.44) |  | 67 (80.72) | 16 (19.28) |  | 55 (80.88) | 13 (19.12) |  |
| **Malnutrition** |  |  | 0.263 |  |  | 0.182 |  |  | 0.825 |
| No | 136 (81.93) | 30 (18.07) |  | 120 (78.95) | 32 (21.05) |  | 75 (82.42) | 16 (17.58) |  |
| Yes | 16 (69.57) | 7 (30.43) |  | 18 (94.74) | 1 (5.26) |  | 25 (80.65) | 6 (19.35) |  |
| **Site** |  |  | 1.000 |  |  | 1.000 |  |  | 0.526 |
| PTB | 142 (80.68) | 34 (19.32) |  | 131 (80.86) | 31 (19.14) |  | 94 (81.03) | 22 (18.97) |  |
| EPTB | 10 (76.92) | 3 (23.08) |  | 7 (77.78) | 2 (22.22) |  | 6 (100.00) | 0 (0.00) |  |
| **Severity of TB** |  |  | **0.002** |  |  | **0.019** |  |  | 0.229 |
| Non-severe | 67 (91.78) | 6 (8.22) |  | 60 (89.55) | 7 (10.45) |  | 31 (88.57) | 4 (11.43) |  |
| Severe | 85 (73.28) | 31 (26.72) |  | 78 (75.00) | 26 (25.00) |  | 69 (79.31) | 18 (20.69) |  |
| **Isoniazid** |  |  | **0.039** |  |  | 0.623 |  |  | 0.221 |
| Non-high dose | 149 (81.87) | 33 (18.13) |  | 128 (80.00) | 32 (20.00) |  | 97 (82.91) | 20 (17.09) |  |
| High dose | 3 (42.86) | 4 (57.14) |  | 10 (90.91) | 1 (9.09) |  | 3 (60.00) | 2 (40.00) |  |
| **Rifampicin** |  |  | 0.052 |  |  | 0.584 |  |  | / |
| Non-high dose | 150 (81.52) | 34 (18.48) |  | 133 (80.12) | 33 (19.88) |  | 100 (81.97) | 22 (18.03) |  |
| High dose | 2 (40.00) | 3 (60.00) |  | 5 (100.00) | 0 (0.00) |  | 0 (0.00) | 0 (0.00) |  |
| **Pyrazinamide** |  |  | 1.000 |  |  | 1.000 |  |  | 1.000 |
| Non-high dose | 147 (80.33) | 36 (19.67) |  | 131 (80.86) | 31 (19.14) |  | 97 (81.51) | 22 (18.49) |  |
| High dose | 5 (83.33) | 1 (16.67) |  | 7 (77.78) | 2 (22.22) |  | 3 (100.00) | 0 (0.00) |  |
| **Treatment** |  |  | **<.001** |  |  | **<.001** |  |  | 0.144 |
| HRZ (E) | 137 (90.13) | 25 (67.57) |  | 130 (84.97) | 23 (15.03) |  | 91 (84.26) | 17 (15.74) |  |
| HRZ (E) + second-line drug (s) | 15 (9.87) | 12 (32.43) |  | 8 (44.44) | 10 (55.56) |  | 9 (64.29) | 5 (35.71) |  |
| **Combination medication** |  |  | **<.001** |  |  | 1.000 |  |  | 0.768 |
| No | 121 (86.43) | 19 (13.57) |  | 121 (80.67) | 29 (19.33) |  | 87 (82.86) | 18 (17.14) |  |
| Yes | 31 (63.27) | 18 (36.73) |  | 17 (80.95) | 4 (19.05) |  | 13 (76.47) | 4 (23.53) |  |
